# Peri‐Implant Soft‐Tissue Phenotype Modification for Refractory Peri‐Implant Mucositis: A 12‐Month Prospective Clinical Study With Ultrasonographic Analysis

**DOI:** 10.1111/jre.70090

**Published:** 2026-02-13

**Authors:** Shayan Barootchi, Lorenzo Tavelli, Hamoun Sabri, Hom‐Lay Wang, Erfan Barootchi, Mario Romandini, Istvan A. Urban, Zoltan Barath

**Affiliations:** ^1^ Department of Oral Medicine, Infection, and Immunity, Division of Periodontology Harvard School of Dental Medicine Boston Massachusetts USA; ^2^ Department of Periodontics and Oral Medicine University of Michigan School of Dentistry Ann Arbor Michigan USA; ^3^ Center for Clinical Research and Evidence Synthesis in Oral TissuE RegeneratION (CRITERION) Boston Massachusetts USA; ^4^ Doctoral School of Medicine University of Szeged Szeged Hungary; ^5^ School of Dentistry Universidad Catolica de Santiago de Guayaquil (UCSG) Guayaquil Ecuador; ^6^ Orthopedic Subspecialty Research Center (OSRC), Sina Hospital Tehran University of Medical Sciences Tehran Iran; ^7^ Ninth People's Hospital Shanghai Jiao Tong University School of Medicine Shanghai China; ^8^ Faculty of Dentistry University of Szeged Szeged Hungary; ^9^ Urban Regeneration Institute Budapest Hungary

**Keywords:** blood circulation, blood flow velocity, crevicular fluid, dental implants, graft, peri‐implant diseases, ultrasonography, wound healing

## Abstract

**Aim:**

This prospective observational study evaluated the clinical, ultrasonographic, and patient‐reported (PROMs) outcomes following surgical treatment of refractory/recurrent peri‐implant mucositis (PM) using implant decontamination combined with soft tissue phenotype modification via a free gingival graft (FGG).

**Methods:**

Subjects presenting with PM unresponsive to two consecutive cycles of non‐surgical therapy—and exhibiting both inadequate midbuccal mucosa width (KMW ≤ 1 mm) and absence of adherent/firm mucosa (AM)—were included. Treatment consisted of an apically positioned flap (APF) with thorough decontamination of implant surfaces and restorative components, combined by placement of a facial FGG. Clinical, high‐frequency ultrasonographic, and PROMs data were assessed at 2 weeks, and at 3, 6, and 12 months post‐operatively.

**Results:**

Twenty subjects with 27 implants were treated. At 12 months, 22 implants (81.5%) were classified as clinically healthy. Significant reductions were observed in bleeding on probing (−85.2%), suppuration (−18.5%), and probing pocket depth (PPD: −0.3 mm). In parallel, significant gains were noted in KMW (+4.42 mm), mucosal recession coverage (+45.7%), and mucosal thickness (MT: +0.87 and 1.04 mm at 1.5 and 3 mm apical to the mucosal margin, respectively). Ultrasonography revealed significantly decreased perfusion parameters and a marked increase in tissue “stiffness” in the coronal facial zone, with 100% of treated sites exhibiting a non‐displaceable band of AM. PROMs showed marked improvements in overall discomfort, discomfort during probing, and overall satisfaction.

**Conclusion:**

Within the limitations of this study, surgical treatment of refractory PM using APF + FGG was effective in disease resolution, achieving reductions in PPD and clinical inflammation, significant gains in MT and KMW, partial recession coverage, and the re‐establishment of a non‐mobile band of AM. Ultrasonographic data confirmed reduced inflammatory perfusion and enhanced soft tissue stiffness in the grafted area, particularly in the most coronal region.

## Graphical abstract

Surgical treatment of refractory peri‐implant mucositis with a free gingival graft (FGG) was effective in disease resolution, achieving reductions in probing pocket depth (PPD) and inflammation. Significant gains in mucosal thickness (MT) and keratinized mucosa width (KMW), as well as partial recession coverage and re‐establishment of attached mucosa (AM) were also noted at 1 year.

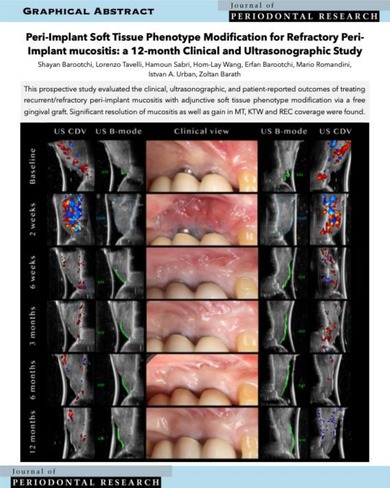

## 1 Introduction

Dental implant therapy has significantly transformed modern dentistry, contributing to improved oral function, esthetics, and overall quality of life for patients [1, 2]. Nonetheless, the growing number of implants placed worldwide has been paralleled by an increasing frequency of implant‐related complications and greater complexity in their management [3, 4, 5, 6, 7]. These complications include lack of osseointegration, poor esthetic outcomes, prosthetic complications, and peri‐implant diseases [8, 9, 10, 11]. Peri‐implant diseases are now commonly encountered in clinical practice [12, 13, 14, 15], with a recent systematic review estimating prevalence rates of 46% for peri‐implant mucositis and 21% for peri‐implantitis [16]. The high prevalence of these conditions is related to their etiology, that is often multifactorial [17, 18]. Several systemic conditions, patient habits, prosthetic, and local factors have indeed been implicated in the development of peri‐implant mucositis and peri‐implantitis [8, 19, 20, 21, 22, 23, 24].

Currently, the treatment of peri‐implantitis is still challenging, and a complete resolution of the inflammatory lesion seems unpredictable [25, 26, 27, 28, 29], with high recurrence rates in the long term [30, 31, 32, 33]. Consequently, there has been a growing emphasis on maintaining peri‐implant health and preventing the onset of peri‐implantitis. In this context, the treatment of peri‐implant mucositis–considered a necessary precursor of peri‐implantitis [34, 35]–has received increased attention. In peri‐implant mucositis, the lesion is still contained within the peri‐implant mucosal compartment, and the surrounding bone has still not been lost [19, 20]. When treated effectively, it presents an opportunity to restore peri‐implant health and prevent disease progression.

The primary etiology for peri‐implant mucositis is biofilm accumulation and the subsequent disruption of the host‐microbe homeostasis at the implant‐mucosa interface, and inflammation is known to be regulated through complex molecular pathways that influence tissue behavior across biological systems [5, 20, 36]. Contributing factors to this condition can include smoking, diabetes mellitus, a history of radiation therapy or periodontitis, prosthetic design issues, poor oral hygiene, inadequate compliance with supportive implant therapy, and the absence of an adequate protective band of soft tissue around the implant [19, 20, 21, 37, 38]. In fact, there is robust evidence supporting a positive role from an adequate band of adherent and firm keratinized mucosa on peri‐implant health and prevention of implant complications [39, 40, 41, 42, 43, 44, 45, 46, 47, 48]. A systematic review and meta‐analysis by Lin et al. found that implants characterized by an adequate band of adherent keratinized mucosa width (KMW) were associated with less plaque accumulation, tissue inflammation, clinical attachment loss, and soft tissue dehiscence, compared to implants lacking or having an insufficient band of adherent KMW [41]. These findings have been echoed in other studies, which suggest that one of the primary benefits of adequate KMW lies in improved patient comfort during brushing and enhanced oral hygiene efficacy [44, 49, 50, 51]. Moreover, a stable band of keratinized mucosa supports vestibular depth, which facilitates access for self‐performed hygiene [19, 40, 42, 52], and may help create a tight soft tissue seal that limits sub‐marginal biofilm colonization.

Thus, it is reasonable to assume that an effective treatment for peri‐implant mucositis at sites lacking an adequate band of adherent KMW should also include soft tissue augmentation procedures aimed at enhancing the adjacent peri‐implant phenotype [53]. Among the different protocols for soft tissue phenotype modification, the apically positioned flap (APF) in combination with a gingival graft (FGG) has shown the most positive results on improving peri‐implant health‐related parameters, in particular in terms of reduction of inflammatory and plaque indices, reduction of the soft tissue dehiscence, and improvement in patient‐reported brushing comfort [43, 53, 54, 55].

Given the limited efficacy of current peri‐implant mucositis treatments in achieving sustained resolution and the risk of progression to peri‐implantitis when left unresolved, the aim of the present study was to evaluate the outcomes of treating recurrent/refractory peri‐implant mucositis with adjunctive APF + FGG therapy.

## 2 Methods

### 2.1 Study Design

The present research was designed as a prospective pre‐post trial, where subjects presenting with recurrent/refractory peri‐implant mucositis (PM) received surgical therapy in the form of peri‐implant debridement and soft tissue augmentation with APF + FGG. The study protocol was approved by the Institutional Review Board of the University of Michigan Medical School (HUM00224580) and was in accordance with the Declaration of Helsinki of 1975, revised in Fortaleza in 2013. All participants were informed and understood the aims and details of the study, and provided written informed consent.

### 2.2 Study Setting and Participants

The study was performed at the clinic of the Department of Periodontology and Oral Medicine, University of Michigan School of Medicine, Ann Arbor, USA between December 2019 and May 2022.

Twenty subjects with either one or two adjacent dental implants with a clinical diagnosis of PM [5, 20] at the time of enrollment, as well as with a recent (less than 6 months from last treatment) history of refractory or recurrent PM which had not resolved after two consecutive cycles of non‐surgical therapy (consisting of mechanical debridement with titanium curettes and ultrasonic instrumentation, supragingival biofilm control, and customized oral hygiene reinforcement), that were characterized by inadequate (≤ 1 mm) or a lack of midbuccal KMW, and absence of adherent/firm mucosa (AM) were included. The definition of the 2017 World Workshop was utilized to define clinical presentation of PM as: (i) the presence of profuse bleeding and/or suppuration on gentle probing (BOP/SUP) with or without increasing probing pocket depth (PPD) compared to previous examinations; and (ii) the absence of progressive bone loss and/or beyond crestal bone level changes resulting from initial bone remodeling.

Other inclusion criteria included: (i) age ≥ 18 years; (ii) functionally loaded implants for at least 36 months; and (iii) subjects willing to receive ultrasound examination for assessing the peri‐implant structures. Exclusion criteria included: (i) subjects taking any systemic medications that could affect the healing of the periodontal and peri‐implant tissues (e.g., phenytoin, calcium channel blockers, cyclosporin, etc.); (ii) subjects reporting smoking habits; and (iii) history of soft tissue grafting at the site of interest, (iv) untreated or unsuccessfully treated periodontal diseases [56, 57].

### 2.3 Intervention—Surgical Treatment With Combined Decontamination and Phenotype Modification

**FIGURE 1:**
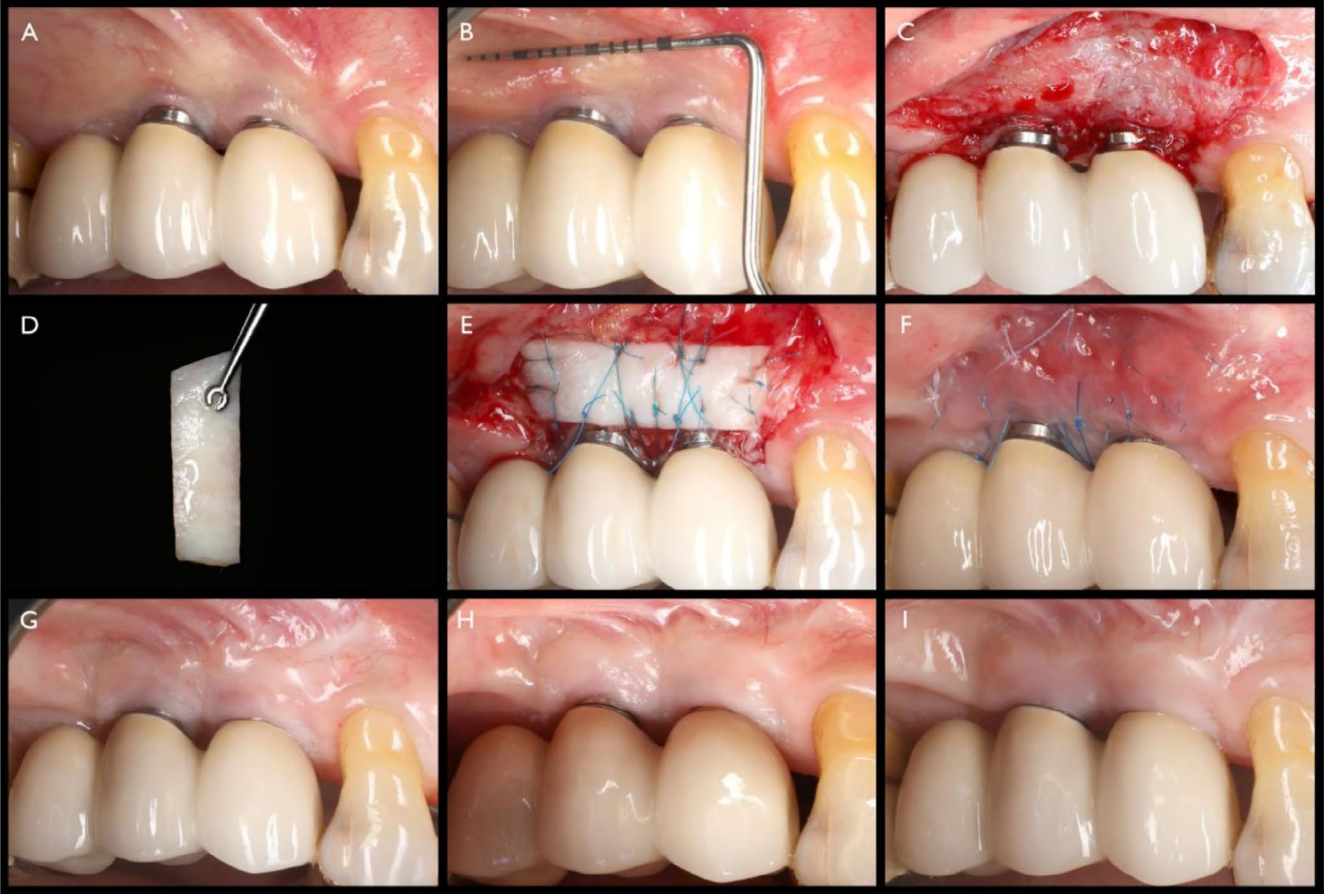
Surgical intervention consisting of an apically positioned flap (APF) on the buccal aspect, in combination with a free gingival graft (FGG) for soft tissue phenotype modification. (A and B) Baseline presentation of implants. (C) Split‐thickness APF preparation, sutured to the periosteum. (D) The harvested FGG. (E) FGG stabilized and sutured. (F) Healing at 2 weeks. (G) Three‐month follow‐up (H) Six‐month follow‐up. (I) Clinical outcome at 1 year.

Professional oral hygiene sessions (non‐surgical therapy) and oral hygiene instructions aimed at improving brushing technique and the overall effectiveness of patients' home care were provided 1 month prior to surgery. It was also planned that patients would be excluded from the study if the condition had resolved by the time of the intervention. The surgical intervention consisted of an APF in combination with a FGG (Figure 1). In the presence of a minimal band of keratinized mucosa the horizontal incision was performed at the mucogingival/mucokeratinized junction, while at sites lacking keratinized mucosa an intrasulcular incision was made [58]. Based on the local characteristics of the site and the presence of adjacent teeth/implants, vertical incision(s) were performed as needed. A split‐thickness flap was elevated and apically positioned approximately 10 mm using T‐mattress sutures (7/0 PGA, AD Surgical, Sunnyvale, USA) anchoring the flap to the periosteum. Care was taken in removing movable soft tissue on the recipient bed.

Thorough decontamination of the implant restorative components and surface (when exposed) was performed using titanium hand curettes and ultrasonic devices [59, 60]. Modifications to the implant suprastructure and/or the implant fixture (implantoplasty) were performed as necessary, on the buccal aspect. The implant supra‐structure was then further cleaned and polished with a rubber cup and a polishing paste, and irrigation with 0.12% chlorhexidine solution (Chlorhexidine Gluconate 0.12%, Peridex, 3M, USA) was performed at the suprabony implant components on the buccal aspect of the implant sites, and into the sulcus on the lingual side. Lastly, the entire surgical site was thoroughly rinsed with sterile saline solution. An epithelialized soft tissue graft (FGG) was then harvested from the hard palate, with the aim of obtaining a graft with a mesio‐distal width slightly less than the recipient bed, and an apico‐coronal height of approximately 6–7 mm, and a thickness of approximately 1.5 mm. After harvesting, the FGG was extra‐orally trimmed to smoothen its margins and remove any excessive adipose tissue from the deeper side of the graft (using a combination of a microsurgical scissor and blade), if necessary. The graft was then stabilized to the periosteum and to the adjacent soft tissues with several simple interrupted sutures (either 7/0 PGA, AD Surgical, Sunnyvale, USA or 7/0 polypropylene [Ethicon, Johnson & Johnson, Somerville, USA], depending on the site). Periosteal anchoring sutures were additionally placed by engaging the periosteum apical to the FGG. The suture was passed lingually beneath the interproximal contact point and looped around the implant‐supported crown, before returning from the lingual to the buccal aspect, where the knot was tied (Figure 1).

A hemostatic collagen dressing (Collatape, Zimmer Biomet, USA) was positioned on the palatal donor site and stabilized through cross‐mattress sutures. Next, cyanoacrylate tissue glue (PeriAcryl 90HV, Glustitch, Delta, Canada) was applied on the collagen sponge, starting from the edges of the harvesting site, moving towards the center, until the tissue glue completely covered the harvested area [61, 62]. A damp gauze was then used to apply firm but gentle pressure on the palate to stabilize the cyanoacrylate tissue glue on the harvested site.

Detailed oral and written post‐operative instructions were given to the patients. Subjects were instructed to avoid mechanical trauma and brushing at the surgical sites for 2 weeks, and to intermittently apply an ice pack for 2–3 times for 20 min during the first 24 h. Patients received 800 mg ibuprofen at the end of the surgical procedure and were instructed to take 600 mg every 6–8 h as needed in the following days. In case of contraindications or allergy to ibuprofen, acetaminophen (500 mg) every 4–6 h was prescribed. Oral systemic antibiotics (amoxicillin 500 mg every 8 h for 1 week) were prescribed to all patients post‐operatively. In addition, all patients received a bottle of an herbal compound rinse (StellaLife VEGA Oral Rinse, StellaLife Inc., IL, USA) and were instructed to use 3 times a day to gently rinse their mouth for the entire two‐week initial healing period.

Subjects returned for a post‐operative visit at 2 weeks, where non‐resorbable sutures were removed (unless indicated), and patients were instructed to resume mechanical tooth cleaning using an extra‐soft post‐surgical toothbrush. Additional follow‐up visits were at 3‐, 6‐, and 12‐month time points from the surgical procedure, where patients also received supportive periodontal and peri‐implant care. Based on the initial healing at 2 weeks and the early outcomes, patients could be provided with another bottle of the herbal compound rinse (StellaLife VEGA Oral Rinse, StellaLife Inc., IL, USA).

### 2.4 Study Outcomes

The primary outcome of the study was resolution of PM at the 12‐month post‐surgical evaluation visit, as assessed clinically [5, 20], followed by ultrasonographic assessment, as a secondary measure of sites. Resolution of peri‐implant mucositis was defined as the complete absence of profuse BOP and absence of SUP at all six aspects of the implant, without an increase in PPD relative to baseline, and with no further radiographic bone loss. Resolution of peri‐implant mucositis was assessed at both the 6‐ and 12‐month follow‐up visits. The primary endpoint was resolution at 12 months, while resolution at 6 months was evaluated as an intermediate (secondary) timepoint.

Additional outcomes included: (i) changes within specific clinical parameters, such as probing depth, KMW, and AMW; (ii) changes within ultrasonographic linear measurements and tissue perfusion outcomes; (iii) changes within the elasticity of the grafted site; and(iv) patient‐reported outcome measures (PROMs).

Three calibration sessions among the participating clinical team members and investigators were conducted (one meeting via Zoom platform, and two clinical sessions). The aim of the meetings was achieving consistency in carrying out the study protocol, assuring a smooth process all throughout, as well as reliability in evaluations. To obtain intra‐examiner reproducibility, training and two separate calibration sessions were held for all the clinical and ultrasonography outcomes until a Kappa statistic of 0.8 or greater was achieved.

### 2.5 Clinical Measurements

The following clinical measurements were obtained at the implant site by a precalibrated operator (S.B.) using a periodontal probe (PCP UNC 15, Hu‐Friedy, Chicago, IL, USA) and rounded up to the nearest 0.5 mm for continuous values: (i) PPD at the mesiobuccal, midbuccal, distobuccal, mesiolingual, midlingual, and distolingual aspects; (ii) presence/absence of profuse BOP, SUP, and plaque, assessed at the mesiobuccal, midbuccal, distobuccal, mesiolingual, midlingual, and distolingual aspects; (iii) midbuccal KMW; (iv) presence/absence of midbuccal AM, clinically determined based on the presence/absence of a band of keratinized tissue that could not be displaced when rolling the soft tissue in an apico‐coronal direction using a periodontal probe, or when probing the implant at the midbuccal aspect; (v) exposure of the suprastructure (MREC), clinically determined as the amount of abutment and/or implant fixture visible on the midbuccal aspect in an apico‐coronal direction, using an acrylic guide (fabricated on the day of the enrollment).

### 2.6 Ultrasonographic Image Acquisition and Linear Measurements

**FIGURE 2:**
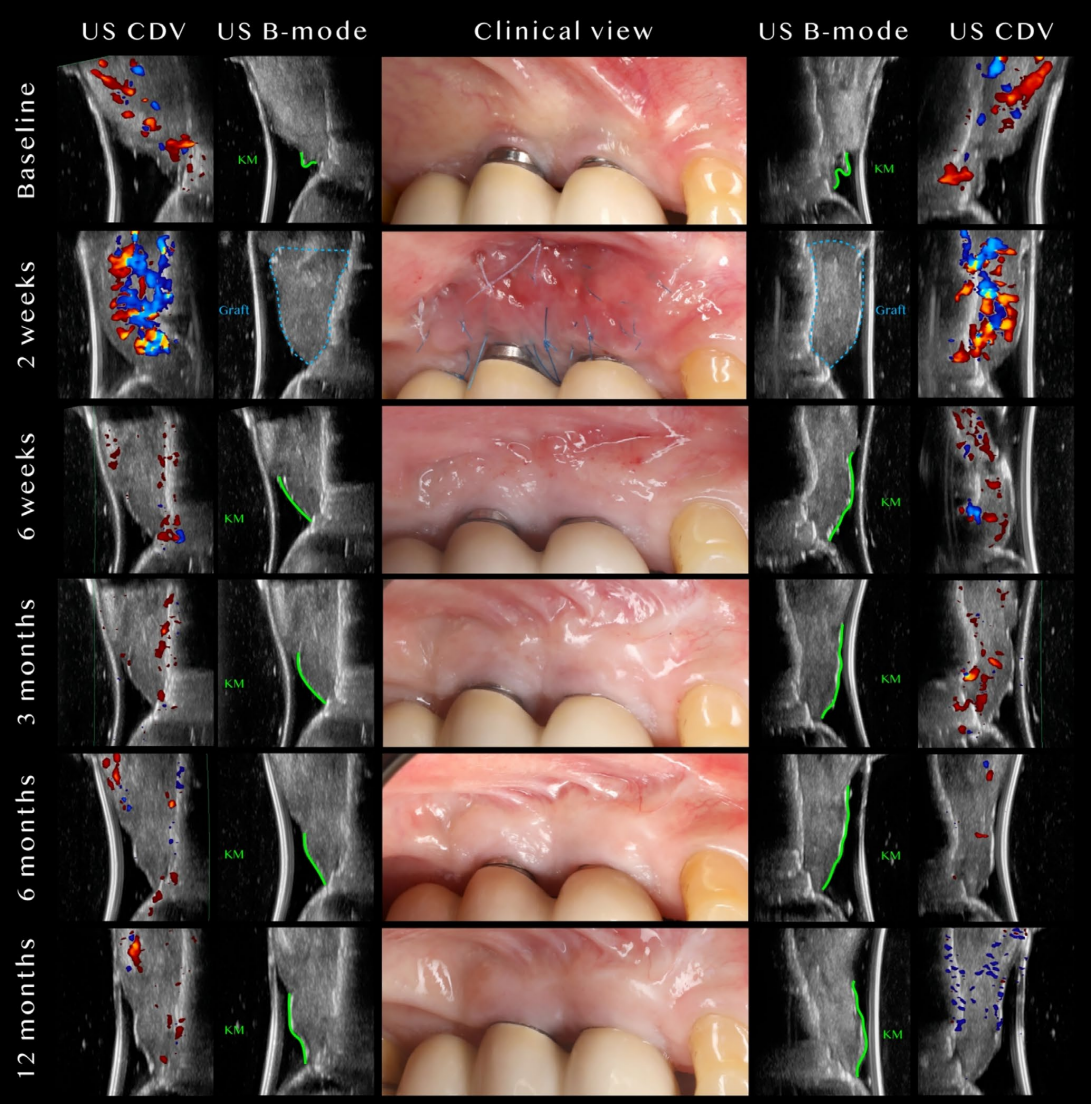
Clinical and ultrasonographic assessment of the healing phenomena occurring at the grafted sites. The first two columns on the left depict the ultrasonographic tissue perfusion, in terms of Color Doppler Velocity (US CDV), and the B‐mode evaluation of the soft tissue changes occurring within the implant #5, while the last two columns on the right illustrate the same outcome measures for the implant #6. The band of keratinized mucosa (KM) is highlighted on the B‐mode ultrasound scans at the different time points.

The ultrasound equipment setup and the scanning procedures have been previously described in detail [63, 64, 65]. Briefly, a commercially available ultrasound imaging device ZS3 (Zonare/Mindray, Mountain View, CA, USA), coupled with a 24‐MHz (64‐μm axial image resolution) and miniature‐sized transducer, was used to obtain ultrasound images (pixel size 0.05 mm). High‐frequency ultrasound (HFUS) B (brightness)‐mode scans were performed by a single operator with extensive expertise in dental ultrasonography at the midbuccal regions of the implant site at baseline (prior to the intervention), and 2‐week, 3‐month, 6‐month, and 12‐month after surgery. B‐mode generates two‐dimensional grayscale images in which brightness is the result of the returned echo signal and its strength, which depends on the acoustical properties of the peri‐implant structures (Figure 2). Then, Doppler ultrasonography modalities were activated to record 6‐s cine loops capturing at least 5 cardiac cycles at the midbuccal and interproximal regions of the implant (Figure 2). Color Doppler Velocity (CDV) and Power Doppler Imaging (PDI) are imaging modalities in which the B‐mode is overlaid with additional color pixels that represent detected blood flow. Color flow (or CDV) imaging is a technique that allows visualization of mean velocity of blood flow within vessels through color coding, based on the scattered signal produced by moving red blood cells that results in a change in frequency of the reflected sound waves that are received by the ultrasound transducer [66]. Color flow provides information on blood flow velocity direction and velocity magnitude, with hues of red and blue colors that are assigned to image pixels based on these two parameters. Blood flow moving towards the transducer is conventionally displayed in red, while blood flowing away from the transducer is usually indicated in blue. PDI is also based on the detection of phase shift changes of the received ultrasound signal. PDI provides information on blood flow quantity that is related to the number of contributing red blood cells that scatter the transmitted acoustic wave. PDI displays in a single‐hue red color the amount of blood flowing within the lumens in the field of view, and it is more sensitive to low flow than color velocity [67, 68].

### 2.7 Ultrasonographic Linear Measurements

The B‐mode and cine loop DICOM files were saved and imported in a commercially available software package (Horos, version 4.0.0, Horos Project).

A precalibrated operator (H.S.) performed the following measurements, as previously described [64, 65, 69, 70, 71]:

- Mucosal thickness (MT): horizontal thickness of the soft tissue, calculated as the distance between the soft tissue margin and the implant surface/buccal bone on a line perpendicular to the long axis of the implant/buccal bone in mid‐buccal scans. MT was measured at 1.5 and 3 mm (MT1.5 and MT3, respectively) from the soft tissue margin.
- Buccal bone dehiscence (BBD): assessed as the vertical distance between the implant platform and the buccal bone crest on mid‐buccal scans.
- Buccal bone thickness (BBT): evaluated 0.5 mm apical to the bone crest as the distance between the peri‐implant crestal bone and a line parallel to the long axis of the implant body on mid‐buccal scans.

### 2.8 Ultrasonographic Tissue Perfusion

**FIGURE 3:**
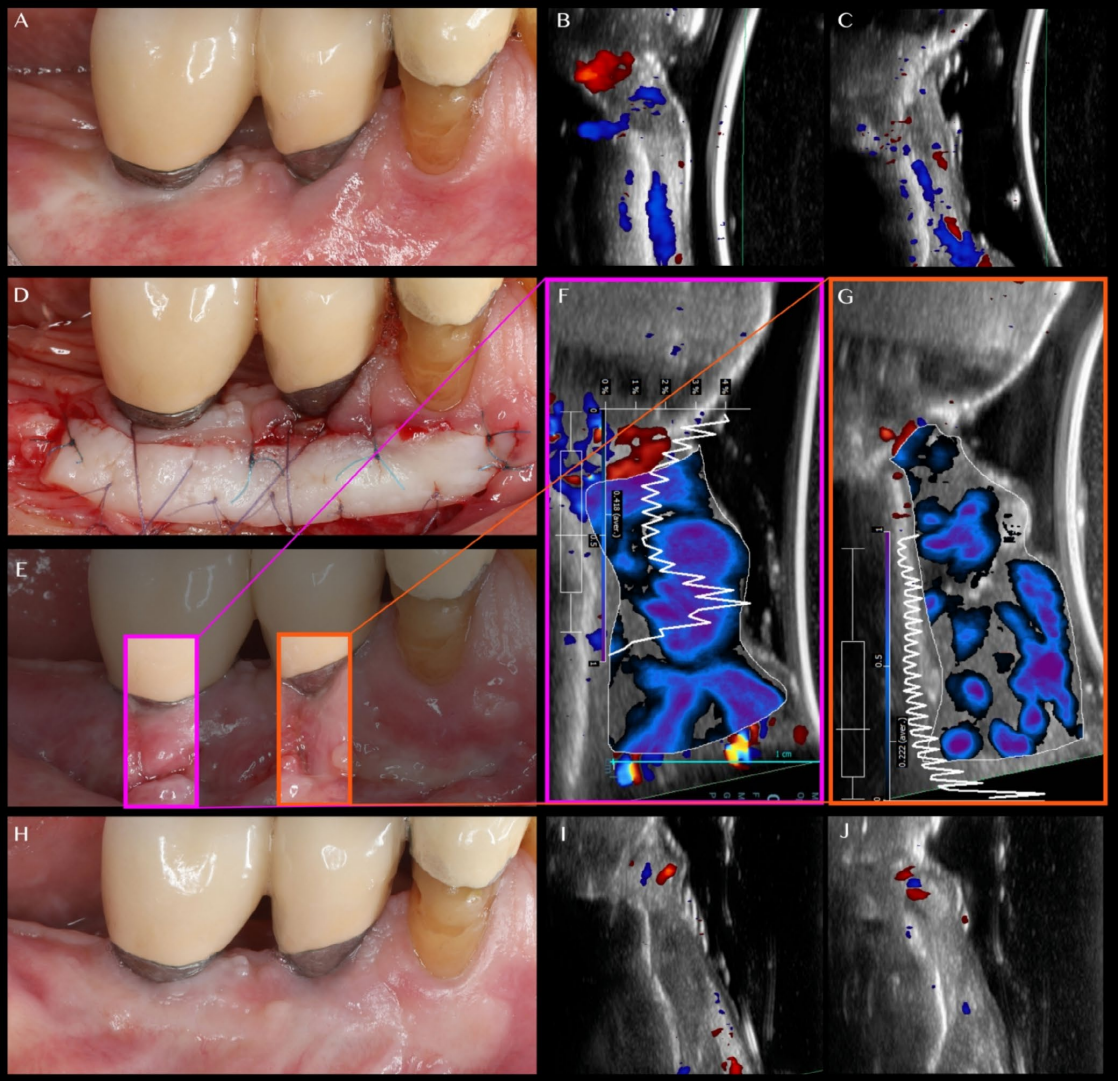
Ultrasonographic tissue perfusion and assessment of perfusion relief intensity (pRI) using the Color Doppler Velocity Mode (CDV). (A) Clinical view at baseline. (B and C) Baseline ultrasound scans of the implants #29 and #30 in CDV mode. (D) Surgical intervention involving APF + FGG. (E) 2‐week follow‐up. (F and G) Ultrasound scans of the implants #29 and #30 obtained in CDV mode at 2 weeks, with the concomitant assessment of pRI of the grafted area. (H) Clinical outcome at 12 months. (I and J) Ultrasound scans of the implants #29 and #30 in CDV mode taken at 12 months.

Tissue perfusion was calculated in terms of CDV and PDI using a software package (PixelFlux, version 2018, Chameleon‐Software, Germany) at baseline, 2 weeks, 3 months, 6 months, and 12 months, as previously described [63, 68, 72, 73, 74, 75] (Figure 3). For both CDV and PDI cine loops, the region of interest (ROI) was defined within the soft tissue as the area between the mucosal margin and extending 7 mm apically to this landmark. The following outcomes were measured in the cine loops recorded in CDV mode:

- Mean perfusion relief intensity (CDV_pRI), which was obtained from the “perfusion relief” function of the software, providing a visual impression of the vasculature depicting the local distribution and intensity of the perfusion within the ROI (Figure 3) [72].
- Flow intensity mix (CDV_FI_mix_), which was calculated by the software with the formula:

$$\mathrm{FI}\;\left(\mathrm{cm}/s\right)=\frac{\text{velocity}\;\left(\mathrm{cm}/s\right)\times A\;\left({\mathrm{cm}}^{2}\right)}{A_{\mathrm{ROI}}\;\left({\mathrm{cm}}^{2}\right)}$$

where velocity corresponds to the color hue of the pixels within the selected ROI, “*A*” is the mean perfused area determined by the number of perfused pixels within the ROI, and *A*_ROI_ is the total area of the ROI.

CDV_FI_mix_ was obtained as the sum of CDV_FI_red_—capturing the perfusion intensity of the blood flowing towards the transducer only—and CDV_FI_blue_, capturing the perfusion intensity of the blood flowing away from the transducer only.

Flow Intensity (PDI_FI) was also calculated on the cine loops obtained in PDI modality, as described above for CDV scans.

### 2.9 Ultrasonographic Tissue Elasticity

**FIGURE 4:**
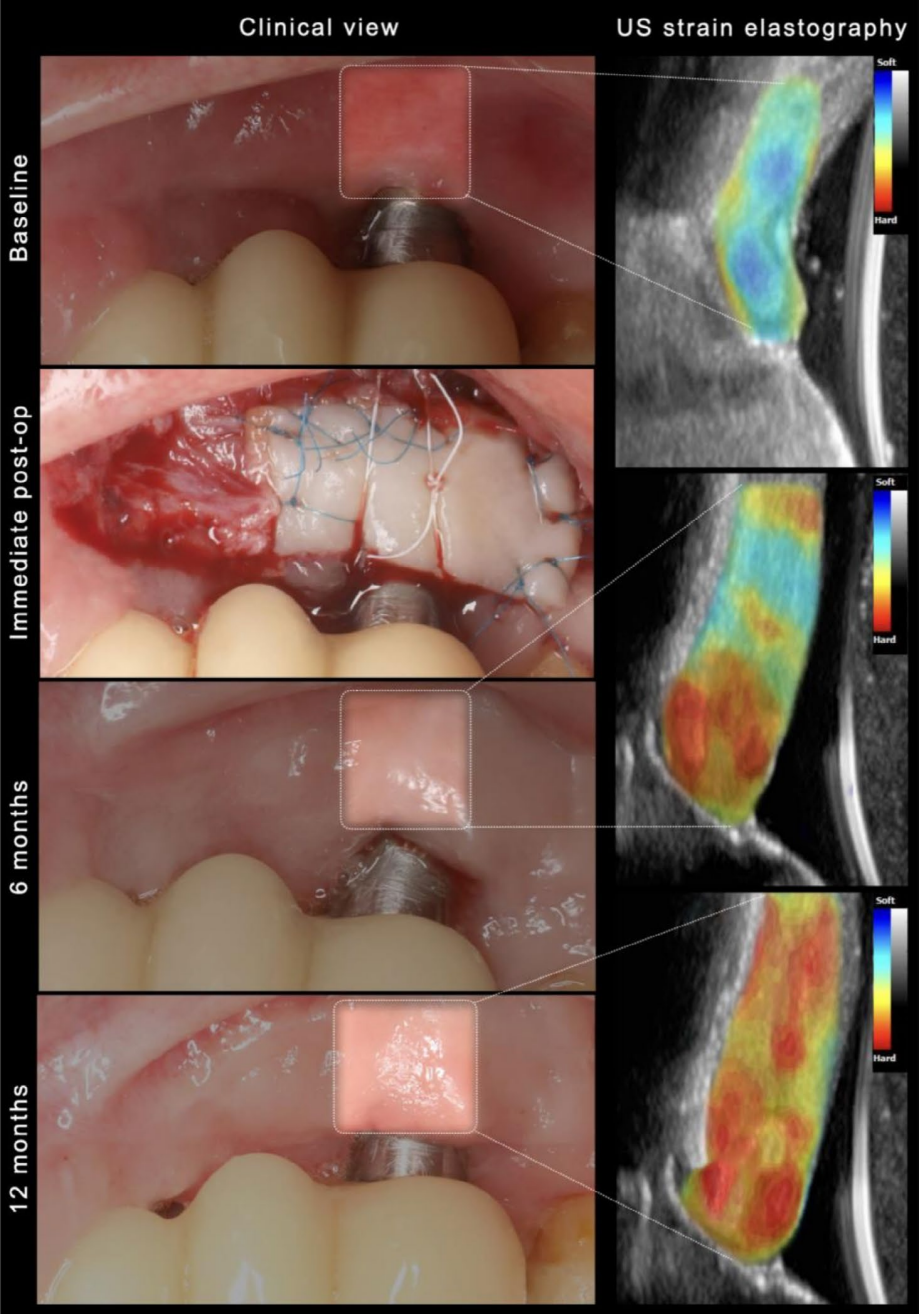
Changes in ultrasonographic (US) tissue elasticity following APF + FGG on an isolated posterior maxillary implant. Orange and red colors in the color‐coded maps (elastograms) indicate that the region of interest is hard/stiff, while colors towards light blue and blue denote that the region of interest is soft/elastic. It is possible to appreciate the increase in stiffness of the grafted area, especially in the coronal portion of the soft tissue, at 6 months and, even more, at 12 months.

The methodology for assessing strain tissue elasticity has been depicted in detail elsewhere [76]. Briefly, the files containing the cine loops with the compression of the tissues were imported in an advanced intelligence elastography software (US‐Elasto, Dileny Technologies and biomedical engineering LCC, Giza, Egypt) that allowed reconstructing strain elastography from gray‐scale imaging videos [77, 78]. The frame‐to‐frame displacement was calculated using a hierarchy recursive displacement tracking technique, with the strain that was computed as displacement spatial derivative and then superimposed on B‐mode scans to visualize anatomical and elastography outcomes using a color‐coded map (elastogram) depicting the different grades of elasticity, from soft to hard (Figure 4) [77]. Two standardized regions of interest (ROIs) were identified within the cine loop video for quantifying tissue elasticity that was then expressed as a strain ratio (SR) [77, 78]. The most coronal portion of the soft tissue (cST) was the first ROI that was identified, while the implant‐supported crown (Cr) was selected as the second ROI, serving as a fixed reference. Then, frame‐to‐frame strain ratio (SR_1_) was calculated by the software with the formula:

$${\mathrm{SR}}_{1}=\frac{{\text{Strain}}_{\mathrm{Cr}}\left(\%\right)}{{\text{Strain}}_{\mathrm{cST}}\;\left(\%\right)}$$

SR_1_ < 1 indicates that the ROI displayed as denominator in the above formula (cST, as the coronal soft tissue) has a higher strain/elasticity and a lower stiffness (rigidity) relative to the other ROI (Cr, as the implant‐supported crown) [79, 80, 81, 82].

In addition, the two standardized ROIs were also positioned at the level of the cST and at the most coronal portion of the alveolar mucosa (AlvM), right below its junction with the above keratinized mucosa (Figure 2), to compute SR_2_ as follows:

$${\mathrm{SR}}_{2}=\frac{{\text{Strain}}_{\mathrm{cST}}\left(\%\right)}{{\text{Strain}}_{\text{AlvM}}\;\left(\%\right)}$$

SR_1_ was computed as StrainCr/StraincST.

SR_1_ < 1 indicates that the coronal soft tissue (cST) exhibits higher strain (i.e., is softer and more elastic) relative to the implant‐supported crown (Cr). Conversely, an increase in SR_1_ reflects a relative stiffening/maturation of the coronal soft tissue.

SR_2_ was computed as StraincST/StrainAlvM.

SR_2_≈1 indicates similar tissue properties between the coronal soft tissue and the AlvM. SR_2_ < 1 indicates that the cST is softer/more elastic than AlvM, while SR_2_ > 1 indicates increased stiffness of the cST relative to AlvM.

From this formula, the difference in tissue elasticity between AlvM and cST was also expressed as:

$${\text{Strain}}_{\text{AlvM}}={{\mathrm{SR}}_{2}}^{-1}\times {\text{Strain}}_{\mathrm{cST}}$$

where ${{\mathrm{SR}}_{2}}^{-1}$ indicates the reciprocal of ${\mathrm{SR}}_{2}$.

SR_1_ was assessed at baseline, 3 months, 6 months, and 12 months, while SR_2_ could be evaluated at the 3‐, 6‐, and 12‐month visits only, when the implant sites displayed a clear demarcation between the cST and the AlvM.

Strain reflects tissue deformation under loading; elasticity describes the ability to return to original shape; stiffness represents resistance to deformation. Lower strain ratio values indicate softer/more elastic tissues, whereas higher values indicate greater stiffness.

### 2.10 PROMs

PROMs were assessed at baseline and at the 12‐month visit using questionnaires with dichotomous questions (yes/no) or using 100‐mm visual analogue scales (VASs) to quantify discomfort at the implant site(s) during daily activities (e.g., during brushing, eating, etc. [DIS]), discomfort when the operator probed the implant site [DIS‐PR] [83, 84], esthetic appearance (EST), and treatment satisfaction (SAT, assessed at the last visit only). Dichotomous questions included “Are you currently satisfied with your dental implant(s)?” and “Would you be willing to undergo the same procedure in the future, if necessary?” (assessed only at the final visit). In addition, patients were asked to fill out a diary during the first four post‐operative weeks capturing the perceived pain after the procedure, using a 0–100 visual analogue scale (VAS). Based on the post‐operative diary, time to recovery, defined as the number of days required to reach a VAS < 10 [65], was computed.

### 2.11 Data Analysis

All data pertaining to the study were combined in a single spreadsheet and de‐identified. Descriptive statistics were utilized to visualize all measures, and the observed data at different timepoints. Means and standard deviations (SD) were calculated for continuous variables. For pairwise comparisons of continuous clinical and PROM variables between baseline and follow‐up time points, paired *t*‐tests or Wilcoxon signed‐rank tests were used as appropriate based on data distribution. For paired categorical outcomes (e.g., resolution of PM at 12 months, presence of BOP/SUP), McNemar's test was applied. All pairwise comparisons were adjusted using Bonferroni correction. To assess changes in ultrasonographic tissue perfusion over time, and the relationships between the ultrasonographic tissue perfusion parameters and clinical outcomes, due to non‐independence of observations, mixed‐effects longitudinal regression models were conducted with random intercepts to account for clustering at the patient level, and time as a fixed effect. Model assumptions were checked, including distribution of residuals and homoscedasticity. A significance level of 5*%* (*α* = 0.05) was assumed for the analyses. The data analysis was performed by an author with experience in management of health‐care data using R (version 4.2.2; R Foundation for Statistical Computing, Vienna, Austria).

## 3 Results

### 3.1 Study Participants Characteristics at Baseline

Twenty subjects (mean age 59.0 ± 18.5 years, 12 females, 8 males) contributing with a total of 27 dental implants were recruited and received the intervention. All subjects completed the appointment visits. Six patients (30.0%) had a history of treated periodontitis, and 3 subjects (15.0%) had controlled diabetes mellitus. Overall, 12 implants were located in the maxilla, while 15 were in the mandible. The mean time in function of the implants was 8.2 ± 4.5 years. Twenty‐one implants were bone level implants, while the remaining 6 were tissue level implants. Eight sites did not show exposure of the abutment/collar or the implant fixture, while 5 implants displayed exposure of the abutment/collar but not of the implant fixture, and 14 implants exhibited exposure of both the abutment/collar and the implant fixture. At baseline, a band of adherent and firm mucosa was missing at all the midbuccal sites, while it was present at the midlingual aspect of 20 implants (100% of maxillary midpalatal sites, whereas absent in 5 of the 15 mandibular implants). The midbuccal MREC was 1.38 ± 1.32 mm, while MT 1.5, MT 3, and BBD were 1.03 ± 0.46, 1.55 ± 0.61, and 3.56 ± 2.11 mm, respectively. Baseline characteristics of the included patients and implants are depicted in detail in Tables 1 and 2.

**TABLE 1:** Characteristics of the study population and implants at baseline.

Parameter	Subjects, sites (*N* = 20, *n* = 27)
Age (mean ± SD) (years)	59.0 ± 18.5
Females (*n* [%])	12 (60)
Subjects with a history of treated periodontitis (*n* [%])	6 (30)
Subjects with controlled diabetes mellitus (*n* [%])	3 (15)
Time of in function of the implants (mean ± SD) (years)	8.2 ± 4.5
Maxillary/mandibular sites [n (%)]	12 (44.4)/15 (55.5)
Implant site (*n* [%])	
Incisors	0 (0)
Canines	2 (7.4)
First premolars	2 (7.4)
Second premolars	5 (18.5)
First molars	12 (44.4)
Second molars	6 (22.2)
Sites not exhibiting exposure of the abutment/collar nor of the implant fixture (*n* [%])	8 (29.6)
Sites characterized by exposure of the abutment/collar only (*n* [%])	14 (51.85)
Sites characterized by exposure of the abutment/collar and the implant fixture (*n* [%])	5 (18.5)
Bone level/tissue level implants (*n* [%])	21 (77.7)/6 (22.2)

Abbreviations: *n*: number; SD: standard deviation.

**TABLE 2:** Clinical and ultrasonographic linear outcomes at baseline, 6, and 12 months.

Parameter	Implant sites (*n* = 27)		
	Baseline	6 months	12 months
Sites diagnosed as clinically healthy/peri‐implant mucositis/peri‐implantitis (*n*)	0 (0%), 27 (100%), 0 (0%)	20 (74.1%), 7 (25.9%), 0 (0%)	22 (81.5%), 5 (18.5%), 0 (0%)
Sites not exhibiting abutment nor implant fixture (*n*, %)	8, 29.6	15, 55.6	18, 66.7
Sites characterized by exposure of the abutment only (*n*, %)	14, 51.9	10, 37.0	8, 29.6
Sites characterized by exposure of the abutment and implant fixture (*n*, %)	5, 18.5	2, 7.4	1, 3.7
Sites with buccal BOP+ (mean) (%) mesial/mid/distal	85.2/92.6/96.3	14.8/11.1/14.8	7.41/7.41/11.1
Sites with lingual BOP+ (mean) (%) mesial/mid/distal	81.5/63.0/85.2	7.4/3.7/11.1	7.4/3.7/11.1
Site with buccal SUP+ (mean) (%) mesial/mid/distal	22.2/18.5/29.6	7.41/0.0/7.41	0.0/0.0/3.7
Sites with lingual SUP+ (mean) (%) mesial/mid/distal	11.1/11.1/22.2	3.7/0.0/7.41	3.7/0.0/3.7
Sites with Plaque (mean) (*n*) buccal/lingual	47/35	10/10	9/12
Buccal PPD (mean ± SD) (mm) mesial/mid/distal	3.27 ± 1.24/2.55 ± 0.97/3.35 ± 1.29	2.98 ± 1.01/2.20 ± 0.77/3.0 ± 0.71	3.00 ± 1.02/2.18 ± 0.77/3.1 ± 0.73
Lingual PPD (mean ± SD) (mm) mesial/mid/distal	3.22 ± 1.15/2.58 ± 1.06/3.41 ± 1.32	3.12 ± 1.11/2.21 ± 0.82/3.09 ± 0.81	3.10 ± 1.09/2.24 ± 0.81/3.08 ± 0.80
Mid‐buccal KMW (mean ± SD) (mm)	0.59 ± 0.57	4.67 ± 1.78	5.01 ± 1.85
Mid‐lingual KMW (mean ± SD) (mm)	2.27 ± 1.58	2.30 ± 1.55	2.26 ± 1.54
Sites with a band of mid‐buccal AM (*n*, %)	0, 0	27, 100	27, 100
Sites with a band of mid‐lingual AM (*n*, %)	20, 74.1	20, 74.1	20, 74.1
MREC (mean ± SD) (mm)	1.38 ± 1.32	0.75 ± 0.44	0.61 ± 0.39
MT1.5 (mean ± SD) (mm)	1.03 ± 0.46	1.89 ± 0.69	1.90 ± 0.68
MT3 (mean ± SD) (mm)	1.55 ± 0.61	2.48 ± 1.06	2.59 ± 1.17
BBD (mean ± SD) (mm)	3.56 ± 2.11	3.59 ± 2.14	3.59 ± 2.13
BBT (mean ± SD) (mm)	1.05 ± 0.54	1.01 ± 0.56	1.02 ± 0.56

Abbreviations: AM: adherent/firm mucosa; BBD: buccal bone dehiscence, assessed as the vertical distance of the crestal bone from the implant platform at the midbuccal aspect; BBT: buccal bone thickness assessed at the midbuccal aspect of the implant; BOP: bleeding on probing; KMW: keratinized mucosa width; MREC: mucosal recession; MT1.5: mucosal thickness assessed 1.5 mm apical to the soft tissue margin; MT3: mucosal thickness assessed 3 mm apical to the soft tissue margin; PPD: probing pocket depth; SD: standard deviation; SUP: suppuration.

Relative to intrasurgical measurements, the recipient bed had a mesio‐distal width of 18.67 ± 6.58 mm, and an apico‐coronal width of 9.49 ± 1.79 mm. The graft width was 17.17 ± 5.86 mm, while its height and thickness were 5.56 ± 0.63 and 1.39 ± 0.24, respectively.

### 3.2 Primary Outcome: Disease Resolution Based on Clinical Assessment

At 6 months, 20 implant sites (74.1%) showed a complete resolution of the disease and were diagnosed as clinically healthy, while 22 implants (81.5%) were diagnosed as clinically healthy at the 12‐month follow‐up.

### 3.3 Clinical and Ultrasonographic Outcomes

The average change in the clinical parameters at the midbuccal aspect of the implants from baseline to 12 months corresponded to an 88.0% reduction in BOP (from 92.6% to 11.1%) and a 100% reduction in SUP (from 18.5% to 0.0%). In contrast, BOP and SUP were consistently more prevalent at interproximal aspects compared with buccal sites at all time points, and suppuration was not completely eliminated at interproximal implant aspects. This site‐specific pattern suggests a differential response to therapy between buccal and interproximal peri‐implant tissues. At the 12‐month visit, the average PPD reduction that was observed at the mesiobuccal, midbuccal, and distobuccal sites of the treated implants, compared to baseline presurgical values, was −0.27, −0.37, and −0.25 mm, respectively.

A significant gain in KMW was observed at 6 months (4.08 ± 1.99 mm) and 12 months (4.42 ± 2.11 mm), compared to baseline. At baseline, none of the implant sites had a band of adherent, firm mucosa at the midbuccal aspect, while at the 6‐ and 12‐month visits, all the treated sites (100%) exhibited a band of adherent mucosa that could not be displaced.

The initial MREC prior to the soft tissue augmentation was, on average, 1.38 mm. At 6 months, the mean MREC was 0.75 mm, indicating that the intervention resulted in a mean MREC reduction of −0.63 mm and in an overall mean MREC coverage of 45.7%. After 12 months, the treated implant sites showed a mean MREC of 0.61 mm, which translates to a mean MREC reduction of −0.77 mm and in an overall mean MREC coverage of 55.8%, compared to baseline. The intervention resulted in a significant gain in MT1.5 (on average, 0.86 mm at 6 months and 0.87 mm at 12 months) and MT3 (on average 0.93 mm at 6 months and 1.04 mm at 12 months) compared to baseline. No significant changes were observed in terms of radiographic marginal bone levels, BBD, nor BBT at any time points (Tables 2 and 3).

**TABLE 3:** Changes within the clinical and ultrasonographic linear outcomes from baseline to 12 months.

Parameter	Changes at implant sites (*n* = 27)	
	BL to 6 months	BL to 12 months
Sites with complete resolution of peri‐implant mucositis, diagnosed as clinically healthy (*n*, %)	20, 74.1	22, 81.5
Average buccal BOP reduction (mean) (%)	−81.5*	−85.2*
Average buccal SUP reduction (mean) (%)	−18.5*	−18.5*
Mesio‐buccal PPD change (mean ± SD) (mm)	−0.29 ± 0.22	−0.27 ± 0.24
Mid‐buccal PPD change (mean ± SD) (mm)	−0.35 ± 0.25	−0.37 ± 0.25
Distobuccal PPD change (mean ± SD) (mm)	−0.36 ± 0.23	−0.25 ± 0.21
MREC reduction (mean ± SD) (mm)	−0.63 ± 0.39*	−0.77 ± 0.42*
Mid‐buccal KMW gain (mean ± SD) (mm)	4.08 ± 1.99*	4.42 ± 2.11*
MT1.5 gain (mean ± SD) (mm)	0.86 ± 0.49*	0.87 ± 0.53*
MT3 gain (mean ± SD) (mm)	0.93 ± 0.56*	1.04 ± 0.65*
BBD change (mean ± SD) (mm)	0.03 ± 0.19	0.03 ± 0.20
BBT change (mean ± SD) (mm)	−0.04 ± 0.17	−0.03 ± 0.15

* Reflects statistically significant change from baseline after Bonferroni adjustment (paired *t*‐test or Wilcoxon signed‐rank test, as appropriate; McNemar test for categorical outcomes; *p* < 0.05).

### 3.4 Tissue Perfusion

Ultrasonographic tissue perfusion outcomes are depicted in detail in Table 4. Overall, during the trajectory of the study timeline (from pre‐treatment to the 12‐month follow‐up), all sites demonstrated a significant reduction of all tissue perfusion parameters (CDV_pRI, CDV_FI_mix_, CDV_FI_blue_, and CDV_FI_red_).

**TABLE 4:** Ultrasonographic tissue perfusion changes over 12 months.

Outcome	Baseline	2 weeks	3 months	6 months	12 months	Estimate [95% CI], *p*
CDV_pRI (mean ± SD) (cm/s)	0.30 ± 0.13	0.38 ± 0.17	0.15 ± 0.13	0.12 ± 0.07	0.13 ± 0.08	−0.004 [−0.005, −0.002], *p* < 0.001
CDV_FI_mix_ (mean ± SD) (cm/s)	0.15 ± 0.07	0.28 ± 0.16	0.09 ± 0.08	0.04 ± 0.06	0.05 ± 0.05	−0.0037 [−0.005, −0.002], *p* < 0.001
PDI_FI (mean ± SD) (cm/s)	0.80 ± 0.23	0.89 ± 0.17	0.59 ± 0.21	0.49 ± 0.22	0.47 ± 0.23	−0.007 [−0.009, −0.004], *p* < 0.001

*Note:* It should be noted that for the PDI modality does not discriminate the direction of the blood flow, and therefore there is no need to perform the sum of red and blue flow intensity (FI) for obtaining the overall value of FI.

Abbreviations: CDV: color doppler velocity; CI: confidence interval; FI_mix_: flow intensity, calculated as the sum of flow intensity red and flow intensity blue; *p*: *p*‐value; PDI: power doppler intensity; pRI: perfusion relief intensity; SD: standard deviation.

Compared to baseline, tissue perfusion increased at the 2‐week time point (0.251 (95% CI [0.173, 0.329]), *p* < 0.001), followed by a progressive decrease later on, reaching values inferior to those observed at baseline. The mean reduction in CDV_pRI from baseline to 12 months was 56.7% (estimate for time being −0.004 (95% CI [−0.005, −0.002]), *p* < 0.001). In addition, baseline perfusion did not correlate with any of the perfusion parameters over time (0.024 (95% CI [−0.24, 0.29]), *p* = 0.85), meaning that the initial inflammation prior to the intervention was not associated with perfusion parameters later on.

The mean reduction for CDV_FI_mix_ (−0.0037 (95% CI [−0.005, −0.002]), *p* < 0.001), and PDI_FI (−0.007 (95% CI [−0.009, −0.004]), *p* < 0.001) overtime also amounted to 66.7% and 41.2%, respectively. Similarly, for both outcomes the 2‐week timepoints were significantly elevated (0.233 (95% CI [0.17, 0.29]), *p* < 0.001), and (0.421 (95% CI [0.28, 0.55]), *p* < 0.001), respectively.

At the 2‐week post‐operative visit, CDV_FI_red_ was found to be significantly higher than CDV_FI_blue_ (0.18 ± 0.07 cm/s vs. 0.09 ± 0.04 cm/s, *p* < 0.001), while no differences between these two parameters depicting blood flow intensity in opposite direction were found at the other time points, suggesting that the main blood supply that FGGs receive during the first 2 weeks of healing is mainly from the periosteum/recipient bed beneath the graft.

### 3.5 Tissue Elasticity

At baseline, SR_1_ (cST/Cr) had a mean value of 0.14 ± 0.09, indicating that the coronal soft tissue (cST) was substantially softer and more elastic than the implant‐supported crown (Cr).

Over time, SR_1_ increased progressively to 0.51 ± 0.19 at 3 months, 0.71 ± 0.18 at 6 months, and 0.84 ± 0.13 at 12 months, a change which was statistically significant over time (0.007 (95% CI [0.005, 0.01]), *p* < 0.001).

As SR_1_ is defined as StrainCr/StraincST, an increase in SR_1_ reflects a relative stiffening of the coronal soft tissue compared to its baseline mechanical behavior. This pattern suggests gradual maturation and increased structural organization of the soft tissues following soft tissue augmentation (Table 5).

**TABLE 5:** Ultrasonographic strain elastography. Changes over 12 months.

Outcome	Baseline	3 months	6 months	12 months	Estimate [95% CI], *p*
SR_1_ (cST/Cr) (mean ± SD)	0.14 ± 0.09	0.51 ± 0.19	0.71 ± 0.18	0.84 ± 0.13	0.007 [0.005, 0.01], *p* < 0.001
SR_2_ (AlvM/cST) (mean ± SD)	NA	0.61 ± 0.19	0.29 ± 0.20	0.21 ± 0.15	−0.008 [−0.01, −0.005], *p* < 0.001

*Note:* SR_1_ was assessed at baseline, 3 months, 6 months, and 12 months, while SR_2_ could be evaluated at the 3‐, 6‐, and 12‐month visits only, when the implant sites displayed a clear demarcation between the cST and the AlvM. SR_1_ was computed as the ratio between Strain_Cr_ and Strain_cST_. SR_1_ values < 1 indicate that the coronal soft tissue portion (cST) has higher strain/elasticity than the crown (Cr). SR2 was computed as the ratio between Strain_cST_ and Strain_AlvM_. SR_2_ values close to 1 indicate that the cST and the AlvM have similar tissue elasticity/stiffness.

Abbreviations: CI: confidence interval; NA: not available; *p*: *p*‐value; SD: standard deviation; SR: strain ratio.

Furthermore, similar to the outcome of tissue perfusion, baseline elasticity did not show to significantly affect future values or results relative to this outcome (0.185 (95% CI [−0.34, 0.71]), *p* = 0.48).

SR2 values were 0.61 ± 0.19 at 3 months, 0.29 ± 0.20 at 6 months, and 0.21 ± 0.15 at 12 months. Given that SR2 = StraincST/StrainAlvM, values < 1 indicate that the cST is softer and more elastic than the underlying alveolar mucosa (AlvM). The progressive and statistically significant reduction in SR2 over time (−0.008 (95% CI [−0.01, −0.005]), *p* < 0.001) indicates increasing stiffness of the alveolar mucosa relative to cST, which is consistent with tissue remodeling, keratinization, and maturation of the augmented mucosa, following APF + FGG. Specifically, the elasticity of the AlvM was 4.76 times the elasticity of the cST at the last study visit.

Overall, both SR1 and SR2 demonstrated directional changes consistent with increased tissue maturity and biomechanical organization over the 12‐month healing period.

### 3.6 PROMs

Prior to the intervention, patients rated DIS 45.3 ± 36.8 VAS, DIS‐PR 33.1 ± 17.5, and EST 39.0 ± 30.4 VAS. At the last visit, these parameters were 14.2 ± 8.7 VAS, 11.5 ± 9.3 VAS, and 54.5 ± 32.6 VAS, respectively. The changes observed for DIS and DIS‐PR were statistically significant (*p* < 0.001, and *p* < 0.01, respectively), while the one related to EST did not reach statistical significance (*p* = 0.212). At baseline 7 subjects (35%) reported an overall satisfaction with their dental implants, while the number of patients stating to be satisfied with their implants at the 12‐month visit was 15 (75%). The SAT rated at the last visit was 89.3 ± 38.3. Nineteen out of 20 subjects (95%) responded that they will be willing to undergo the same intervention in the future, if necessary. The mean perceived post‐operative pain during the first 2 weeks after surgery was 37.8 ± 25.9, while the average time to recovery was estimated to be 12.9 days (Table 6).

**TABLE 6:** Patient‐reported outcome measures (PROMs) assessed at baseline, 12 months, and during the first post‐operative month.

Time of assessment	PROM	Subjects (*N* = 20)
Baseline	DIS (mean ± SD) (VAS point 0–100)	45.3 ± 36.8*
	DIS‐PR (mean ± SD) (VAS point 0–100)	33.1 ± 17.5^#^
	EST (mean ± SD) (VAS point 0–100)	39.0 ± 30.4
	Subjects satisfied with the dental implant(s) (*N*)/(%)	7 (35)
12 months	DIS (mean ± SD) (VAS point 0–100)	14.2 ± 8.7*
	DIS‐PR (mean ± SD) (VAS point 0–100)	11.5 ± 9.3^#^
	EST (mean ± SD) (VAS point 0–100)	54.5 ± 32.6
	SAT (mean ± SD) (VAS point 0–100)	89.3 ± 38.3
	Subjects satisfied with the dental implant(s) (*N*)/(%)	15 (75)
	Subjects willing to undergo the same procedure in the future, if necessary (*N*)/(%)	19 (95)
Post‐operative phase	Pain during the first 2 weeks (mean ± SD) (VAS point 0–100)	37.8 ± 25.9
	Overall pain during the first 1 month (mean ± SD) (VAS point 0–100)	17.1 ± 27.3
	Time to recovery (mean ± SD) (days)	12.9 ± 3.0

*Note:* * and # reflect statistically significant changes from baseline to 12 months (paired *t*‐test or Wilcoxon signed‐rank test with Bonferroni correction), in each respective outcome.

Abbreviations: DIS: discomfort in daily activities (e.g., during brushing, eating, etc.); DIS‐PR: discomfort when the operator probed the implant site; EST: esthetic appearance; SAT: treatment satisfaction; SD: standard deviation; VAS: visual analogue scale.

## 4 Discussion

The primary goal of this investigation was to assess the efficacy of a surgical approach with inclusion of soft tissue phenotype modification with APF + FGG to treat implant sites exhibiting recurrent or refractory mucositis. Overall, this approach showed promising results within its first 12 months, as demonstrated by a complete resolution of the inflammatory condition in 81.5% of the sites.

The pathophysiology of peri‐implant mucositis is multifactorial and influenced by microbial, prosthetic, and soft‐tissue factors [20, 60, 85, 86, 87]. Among these, the configuration of the peri‐implant mucosa plays a central role in determining susceptibility and responsiveness to therapy [19, 20, 85]. To this aim, the present study included only symptomatic diseased dental implants characterized by an inadequate amount of mid‐buccal KMW which lacked a band of adherent and firm AM. Although peri‐implant mucositis has been conventionally treated with non‐surgical therapy [19, 42, 60, 85], a clear consensus on the most effective decontamination protocol has not yet been reached [85, 87, 88]. It is reasonable to assume that, when flap elevation is performed within the context of soft‐tissue phenotype modification, it may also allow access for the decontamination or modification of cement‐retained suprastructures, as well as for the detection and removal of excess cement [21, 89, 90, 91].

With these advantages in mind, clinicians should also be aware that raising a flap around a diseased implant may also lead to a soft tissue dehiscence or worsening of a pre‐existing implant esthetic complication, especially when the flap is entirely composed by alveolar mucosa. It should also be clarified that flap reflection in the present study was not performed for sole the purpose of obtaining access to the implant surface. The surgical approach was undertaken to correct the unfavorable soft‐tissue phenotype—specifically, the complete absence of buccal KMW and the presence of mobile alveolar mucosa—which can predispose to persistent inflammation and reduced responsiveness to non‐surgical therapy. As such, an APF + FGG in these scenarios may in fact be a suitable approach—specifically at non‐esthetic sites—as this treatment modality facilitates decontamination and modification of the suprabony components of the implant and addresses one of the main risk indicators associated with inflammation, plaque accumulation, and inability to perform proper oral hygiene (i.e., an inadequate KMW and lack of AM) [19, 37, 41, 42, 43]. Indeed, a study by Monje and Blasi reported that almost 96% of dental implants with less than 2 mm of KMW exhibited an inadequate vestibular depth [42], which led to the implants being at a greater risk for developing or progressing MREC, clinical attachment loss and radiographic bone loss [52]. Furthermore, Grischke et al. showed that a lack of an adequate amount of KMW was correlated to the severity of peri‐implant mucositis [40].

Recent studies have also demonstrated that a peri‐implant soft tissue phenotype that is characterized by less than 2 mm of KMW is detrimental to the resolution of peri‐implant mucositis after non‐surgical therapy [86, 87, 92]. In a study by Fons‐Badal et al., the authors found an initial 83% resolution of peri‐implant mucositis at 1 month after non‐surgical therapy for implants without KMW, which later relapsed and was significantly reduced at 3 and 6 months, with only 27% of the implants that remained clinically healthy [87]. Conversely, implants with KMW maintained the early outcomes of non‐surgical therapy up to 6 months, with an overall resolution rate of 90%. In addition, the same study also found that KMW positively affected the stability of PPD reduction after non‐surgical therapy, with implants lacking KMW showing a relapse in PPD at 6 months, leading the authors to conclude that a band of KMW is necessary to provide stability to treatment outcomes and to the adjacent soft tissues [87]. Similar findings were also reported by Blasi and coworkers, who obtained a complete resolution of peri‐implant mucositis 6 months after non‐surgical therapy in 50% of implants presenting with ≥ 2 mm of KMW, while only in 16.7% of sites that showed < 2 mm of KMW [86]. The authors found that a narrow KMW was associated with reduced odds of disease resolution by 80% compared to an adequate band of KMW [86].

Considering these results from a different perspective, most of the studies published on non‐surgical treatment of peri‐implant mucositis have all failed to show a complete resolution of the inflammatory condition, even in a compliant cohort. Indeed, in our study, the group of included subjects had all been part of a regular maintenance interval program and attended recall appointments that included supportive implant therapy. Regardless of this fact, and the notion that these patients were all clinically “complaint” (as they actively adhered to and underwent repeated sessions of non‐surgical therapy), many implant sites had not shown a complete resolution of the inflammatory condition and thus were recruited as part of our research.

This phenomenon raises speculations. One being that the etiological factors of peri‐implant diseases, whatever it/they may be, may not be effectively controlled or eliminated via non‐surgical therapy alone. Another hypothesis is that the term “compliance” if it only reflects the yearly number of cleaning visits patients adhere to, may simply not be enough in the context of assessing patients' quality of at‐home hygiene care and plaque removal. Another plausibility can be the notion that there is still no exact evidence as to how often and how approximate patients' cleaning visits should be, as to avoid and/or monitor and treat implant diseases, and for maintaining implant health. Nevertheless, considering the relatively superior (while not yet pristine) results of our study compared with previous research on the resolution of PM, it is reasonable to assume that lower outcomes can be associated with the limitations of non‐surgical therapy (which can in part be overcome with access flap therapy) and/or to the persistence of etiological factors and risk indicators that contribute to the disease. Our results show an overall satisfactory outcome when APF + FGG was performed at implant sites with refractory peri‐implant mucositis and lack of adequate KMW and AM. The clinical outcomes showed to be stable (or even improve) over 12 months. The gain in KMW was, on average, 4.08 mm at 6 months and 4.42 mm at 12 months, with all the treated sites exhibiting a band of adherent and firm AM after the intervention. Although reducing the depth of the pre‐existing MREC was not a primary goal of this intervention, APF + FGG was also able to provide a stable level of the soft tissue margin and even promote a partial coverage of dehiscences (up to 55.8% of MREC coverage) at 12 months.

As it relates to the clinical outcomes achieved with the FGG, the magnitude of KMW gain, the recreation of a band of adherent AM, the stability of the soft tissue margin (with partial coverage of the MREC), and the significant reduction of inflammatory indices are in line with previous studies assessing the outcomes of APF + FGG at implant sites [39, 43, 53, 55, 93, 94]. Among the uniqueness of the present research lies the non‐invasive and real‐time assessment of wound healing events and longitudinal assessment of soft tissue phenotypic modification at the grafted sites using HFUS. Over the last 5 years, our group has extensively explored the benefits of adopting ultrasonographic assessments for clinical research around teeth and dental implants [63, 68, 95, 96]. First, this technology allowed for real‐time quantification of the changes that occurred in the soft tissue phenotypic as it relates to gain in mucosal thickness, without the need to perform local anesthesia or exposure of patients to radiations. Specifically, we found an average gain of MT1.5 and MT3 of 0.87 and 1.04 mm at 12 months, respectively. The assessment of these parameters is crucial given the role of MT on peri‐implant health, esthetics, and on the stability of the soft tissue margin over time [19, 97, 98]. In addition, by being able to assess buccal bone levels after the surgical therapy, we did not find a significant change within the first year at the level of buccal bone, in terms of BBD and BBT. The assessment of these variables has rarely been reported in the literature when it comes to soft tissue phenotype modification using APF + FGG [53, 54, 99]. In addition, our ultrasonographic tissue perfusion analysis illustrated how vascularity evolved over the healing period. Perfusion values increased at the 2‐week time point—consistent with the expected hypervascular phase of graft healing—and progressively decreased thereafter at the 3‐, 6‐, and 12‐month evaluations. Although previous studies from our group and others have shown correlations between ultrasonographic perfusion metrics and clinical or histologic markers of inflammation, since the tissues examined at each time point differed in their anatomical composition (native mucosa at baseline, a healing FGG during the early post‐op period, and a mature keratinized graft at 12 months), these perfusion trends should be interpreted primarily as changes in vascularity rather than as direct indicators of inflammation. Thus, within the limitations of the current knowledge, our findings reflect perfusion dynamics during healing and soft tissue maturation. It should also be noted that among the parameters of our assessed ultrasonography, CDV and PDI provide complementary information: CDV quantifies both flow direction and velocity, whereas PDI detects low‐velocity microvascular perfusion and is more sensitive to early inflammatory changes. CDV_pRI captures spatial distribution of perfusion, while CDV_FImix and its red/blue components quantify vector‐specific flow dynamics. The combination of these metrics provides a more complete biologic characterization of peri‐implant inflammation and graft revascularization.

Interestingly, the grafted area at 2 weeks exhibited a significantly higher flow intensity from the periosteum/bone towards the transducer than the other way around, which is in line with classical studies depicting FGG revascularization [100, 101]. Ultrasonographic tissue elasticity analysis demonstrated for the first time the effect of FGG on the intrinsic characteristics of the peri‐implant soft tissue, that becomes significantly stiffer after the intervention. This innovative analysis, that has been recently introduced in the soft tissue arena by our group for the evaluation of CTG‐based soft tissue augmentation procedures [76], also revealed that FGG undergoes a continuing maturation/remodeling process within the first 12 months, resulting in a progressive increase in tissue stiffness. It can be speculated that this process may also be related to the phenomenon of creeping attachment, that is known to be a prerogative of autogenous FGG.

Within the limitations of the study, the lack of a comparative study arm, the relatively short follow‐up, and the inclusion of implants with inadequate KMW and lack of AM only should be mentioned. It would have been beneficial to have one or more additional study arms involving the use of APF alone, APF + the strip gingival graft technique, or bilaminar techniques with autogenous graft or substitutes. In addition, due to the small number of non‐resolved sites, further analysis could not be performed to evaluate baseline perfusion metrics as a prognostic indicator. Future studies with larger cohorts are needed and underway for this endeavor.

Biomarkers‐assisted assessment of early wound healing events following APF + FGG would have also provided additional mechanistic insights that could have been integrated to the ultrasonographic findings. Future studies with additional study arms, larger cohorts, longer follow‐ups, and additional technologies are encouraged.

## 5 Conclusions

The present clinical and ultrasonographic study evaluated the outcomes of mechanical instrumentation combined with soft tissue augmentation using APF + FGG within the context of a surgical management of refractory peri‐implant mucositis. At 12 months, 81.5% of treated sites displayed complete resolution of the condition. All sites exhibited a significant gain in KMW and a successful re‐establishment of a band of AM. The intervention also led to reductions in PPD, gains in MT, and partial MREC coverage. Grafted sites showed a progressive increase in soft tissue stiffness, particularly in its most coronal portion, as well as significant reductions in ultrasonographic tissue perfusion parameters. PROMs revealed significant improvements in overall discomfort and discomfort during probing, and satisfaction with the treated implants.

## Author Contributions

S.B. and L.T. contributed to study conception and design, data acquisition, analysis and interpretation, and manuscript drafting. H.S. contributed to study conception, data acquisition and analysis, and manuscript drafting. H.‐L.W. contributed to manuscript drafting, data interpretation, and critically revised the manuscript. E.B. contributed to data acquisition and analysis and manuscript drafting, and critically revised the manuscript. M.R. contributed to data interpretation and critically revised the manuscript. I.A.U. contributed to study conception and manuscript drafting. Z.B. contributed to study conception, data interpretation, and critically revised the manuscript.

## Funding

The study was supported by a researcher grant from the Osteology Foundation (Project No. 21‐169), and by a grant from the Delta Dental Foundation (AWD010089). The authors do not have any financial interests, either directly or indirectly, in the products or information listed in the paper.

## Disclosure

This manuscript used NLP (ChatGPT) for proofreading specific sections of an entirely human‐generated text. No other use of AI was made.

## Conflicts of Interest

The authors do not have any financial interests, either directly or indirectly, in the products or information enclosed in this manuscript. Mario Romandini serves as Editor‐in‐Chief and Shayan Barootchi as Associate Editor of the *Journal of Periodontal Research*, and they are also authors of this article. In accordance with Wiley's standard policies for submissions by Editors, they were excluded from the editorial decision‐making and remained blinded throughout the peer review process, with another journal Editor designated as acting Editor‐in‐Chief.

## Data Availability

The data that support the findings of this study are available from the corresponding author upon reasonable request.
